# Emergency Physician Reduction of Pediatric Hip Dislocation

**DOI:** 10.5811/cpcem.2019.1.41131

**Published:** 2019-02-26

**Authors:** Seth Capehart, Brenden J. Balcik, Rosanna Sikora, Melinda Sharon, Joseph Minardi

**Affiliations:** * West Virginia University School of Medicine, Morgantown, West Virginia; † West Virginia University School of Medicine, Department of Emergency Medicine, Morgantown, West Virginia; ‡ West Virginia University School of Medicine, Department of Medical Education, Morgantown, West Virginia

## Abstract

Traumatic hip dislocation in children is relatively rare but presents a true emergency, as a delay in reduction can result in avascular necrosis of the femoral head and long-term morbidity. After sustaining a traumatic posterolateral hip dislocation, a seven-year-old boy presented to an outside facility where no attempt was made at reduction. The patient was transferred to our emergency department (ED) where he was promptly sedated and the dislocation was reduced in a timely manner. Emergency physicians have demonstrated high success rates with dislocation reduction. ED reduction should occur immediately to reduce the likelihood of long-term complications. While timely consultation with a pediatric orthopedist is recommended, that should not delay reduction. The reduction should ideally be performed before the patient leaves the department or is transferred to another facility.

## INTRODUCTION

Traumatic hip dislocation in pediatric patients is relatively rare, with little literature to support specific treatment techniques and timelines. When compared to adults, pediatric hip reduction may be easier to perform and carries a more favorable prognosis.^1^ A delayed reduction may result in avascular necrosis (AVN) of the femoral head. Early-stage AVN is usually painless but ultimately leads to painful degradation of the hip joint, restricted movement and eventual collapse, requiring arthroplasty.^2^ In this report, we describe a seven-year-old boy with a traumatic posterolateral hip dislocation presenting to an emergency department (ED). In addition, we discuss the current literature and review the reduction procedures, so that subsequent cases may be treated as early as possible to decrease potential morbidity from this uncommon injury in the pediatric population.

## CASE REPORT

A seven-year-old boy presented to an outside facility after sustaining a hip injury while playing organized football. He reported that he was struggling at the bottom of a pile when he developed severe hip pain. At the outside hospital, he was diagnosed with a posterolateral hip dislocation. No attempt at reduction was made at the referring hospital. After consultation with a local orthopedist, the patient was transferred to our tertiary care facility via helicopter, and he arrived approximately 5.5 hours after initial injury. Prior to his arrival, preparations were made by ED staff for a rapid, comprehensive trauma evaluation and emergent sedation and reduction measures.

**Image 1:**
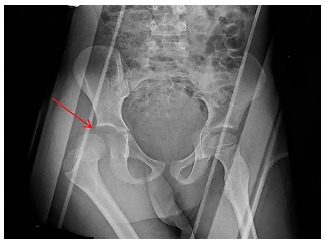
Anterioposterior pelvic radiograph demonstrating superior and lateral displacement of the right femoral head relative to the acetabulum (arrow) and internal hip rotation consistent with posterior hip dislocation.

The patient complained of right leg pain and tingling upon arrival, with no reported pain elsewhere. Physical exam revealed that he was moderately distressed from pain and slightly tachycardic. The right lower extremity was internally rotated with flexion at the knee. He had normal distal pulses, good capillary refill, and was able to move his toes. Pelvic radiograph confirmed posterolateral right femoral head dislocation without evidence of fracture, as seen in Image 1. Point-of-care focused assessment with sonography for trauma exam and chest radiograph were also completed and both were negative.

**Image 2:**
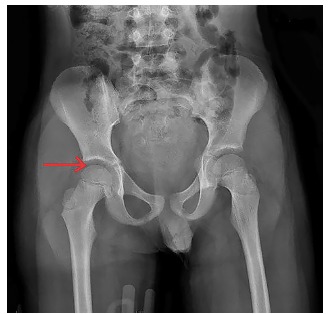
Post-reduction anterioposterior view of the pelvis demonstrating normal alignment of the right femoral head within the acetabulum (arrow).

A complete trauma evaluation confirmed an isolated right hip dislocation with no contraindications to procedural sedation to facilitate dislocation reduction. Given his stable hemodynamics, he was sedated with intravenous propofol. Once sedated, the pelvis was stabilized by providing posteriorly directed countertraction to the pelvic girdle preparing for reduction via Allis technique. The emergency physician (EP) stood on the bed, flexed the hip and knee to 90 degrees, placing the patient’s right leg into a simulated seated position, and provided steady anterior traction by pulling from behind the knee and slightly internally rotating. The right hip was easily reduced without complication and the patient remained hemodynamically stable. Post-reduction, radiographs were performed showing complete reduction of the femoral head, as seen in Image 2. The patient’s pain was improved and his paresthesias were resolved.

**CPC-EM Capsule**

What do we already know about this clinical entity?

*Posterolateral femoral head dislocations in the pediatric population are relatively rare and are often referred to surgery for reduction*.

What makes this presentation of disease reportable?

*While a relatively rare injury, pediatric hip dislocations are a true emergency due to risk of femoral head avascular necrosis resulting in increased morbidity*.

What is the major learning point?

*Reduction procedures should be performed by the initial treating emergency physician (EP), in a timely manner, to decrease the risk of avascular necrosis of the femoral head*.

How might this improve emergency medicine practice?

*Recognizing the risks of delayed reduction, the techniques, and safety of closed reduction should allow EPs to more rapidly treat these injuries leading to improved outcomes*.

Pediatric orthopedics was subsequently consulted, recommending a pelvic computed tomography (CT), which was negative for fracture. The patient was placed in a knee immobilizer and admitted for observation. He was discharged the next day with a walker, restricted to toe-touch weight-bearing of the right lower extremity with no hip flexion past 90 degrees or adduction past midline. He did well in follow-up, returning to full weight-bearing activity and exercise two months post-injury.

## DISCUSSION

Although pediatric hip dislocation is relatively rare, it is a time-sensitive diagnosis. Delays in reduction result in increased risk of morbidity, namely AVN.^3^ EPs must be knowledgeable about and properly trained to diagnose and treat this condition. In children aged 6–10 years, the most common cause of injury is the result of a minor impact such as a fall from height or during an athletic event. Children 10 years of age and older mostly have hip dislocations due to a motor vehicle collision.^1^ Approximately 80% of these types of pediatric injuries result in posterolateral dislocation as a result of minimal trauma. Dislocation in children is attributed to the ligamentous laxity and a soft, pliable acetabulum, which also accounts for a low incidence of associated acetabular and femur fractures compared to adults.^4^ The predominant mechanism of injury is posteriorly directed force along the femoral shaft while the hip is held in flexion, adduction, and internally rotated.^5^

Physical exam findings of posterior dislocation include prominent, elevated greater trochanter and a shortened, internally rotated, flexed, and adducted lower extremity. Conversely, anterior dislocation presents with a loss of greater trochanter prominence in an externally rotated, extended, and abducted lower extremity, with possible leg lengthening.^5^ Superior lateral displacement of the femoral head with respect to the acetabulum is consistent with posterior dislocation, while anteromedial displacement is consistent with anterior dislocation.^6^–^8^ Children who suffer minor trauma and refuse to weight bear should be carefully examined for findings suggestive of posterior hip dislocation, in addition to a full evaluation for other traumatic injuries as well as peripheral nerve injury.^6^

Diagnosis is confirmed radiographically and should be ordered immediately so that reduction can be performed within six hours from time of injury. In a longitudinal study, Mehlman et al. found a 20-fold increase in AVN of the femoral head for pediatric patients having reduction performed after six hours, while 95% of patients who underwent reduction in less than six hours had favorable outcomes.^3^ Additionally, a meta-analysis comparing AVN rates for early (< 6 hours) and late reductions (> 6 hours) from five eligible studies showed a significant, decreased risk of AVN to those undergoing early reduction.^9^ Other potential complications of pediatric hip dislocation include sciatic nerve injury, coxa magna (a usually asymptomatic rounding and shortening of the femoral head and neck), re-dislocation, and post-injury arthritis. In adolescents, one must consider and evaluate for epiphysolysis (complete separation of the proximal femoral epiphysis), which leads to AVN in nearly all cases. CT or magnetic resonance imaging (MRI) may be necessary to establish this diagnosis post-reduction, but the optimal imaging modality has not been established.^6^

Management of injury includes prompt pain management, sedation or general anesthesia, emergent reduction of dislocated hip and post-reduction immobilization or traction. While the majority of the literature describes reductions that occur in the operating room (OR), reductions can safely be performed in the ED, allowing for a shorter interval between dislocation and reduction and reducing the risk of AVN. Existing literature suggests that all reductions for hip dislocations, including pediatric and even prosthetic hip dislocations, can be safely and effectively carried out in the ED.^6^,^10^ In a systematic review by Bressan et. al., which included 25 case reports and case series, nearly half of reported acute cases were reduced in the ED with no reported adverse outcomes.^6^ All cases of reported AVN were associated with delayed reduction (> 6 hours) performed in the OR, which would suggest that early reduction in the ED by competent providers is protective against AVN.^6^ This review was unable to identify an optimal reduction technique.^6^ This case demonstrates an additional example of safe and successful closed pediatric hip reduction performed in the ED.

A variety of reduction techniques have been described and are summarized in the Table reproduced from Gottlieb, stating that the individual success rates vary from 60–90% across all age groups.^11^ The best technique for pediatric reduction has not been identified and should be based on the experience of the providing physician.^6^ Therefore, EPs should be familiar with several different techniques to increase the odds of success.^11^ Early consultation with pediatric orthopedic surgery is recommended and patients should be followed and examined for resulting AVN and/or growth disorders.^6^,^12^

## CONCLUSION

Although a relatively rare injury, pediatric hip dislocations do occur and the ED should be the primary location for closed reduction without evidence of fracture. In younger children these injuries are more often due to lower energy trauma, and associated fractures are less common than in older children and adults. EPs should be familiar with the diagnosis and reduction procedures to reduce the risk of long-term complications, namely AVN, and financial cost to patients. Closed reduction should not be delayed pending transfer to an alternate facility, consultation with pediatric orthopedics, or availability of an OR. Reduction should be performed as soon as possible, preferably within six hours of dislocation to decrease the risk of AVN of the femoral head.

## Figures and Tables

**Table:** Review of techniques for hip dislocation reduction by Gottlieb.^11^

Name	Technique	Advantages	Disadvantages
Allis	Provider grasps affected leg with both knee and hip flexed to 90 degrees, applying traction toward the ceiling.	Well-established	Risk of falls and lower back injury to the provider.
Bigelow	Provider grasps affected leg with both knee and hip flexed to 90 degrees, applying in-line traction while abducting, externally rotating, and extending the leg.	This technique is no longer recommended.	Risk of falls and lower back injury to the provider. Increased risk of femoral neck fractures.
East Baltimore lift	Two providers place their arms underneath the affected knee with their knees bent and their hands on each other’s shoulders. Providers slowly stand up while countertraction is applied to the patient’s ankle.	Strong, controlled upward force and ability to internally and externally rotate the hip	Requires multiple providers.
Tulsa/Rochester/Whistler	Provider places the arm underneath the affected knee with the provider’s palm on the flexed, unaffected knee. Using the forearm as a fulcrum, the provider applies downward pressure on the ankle, while internally and externally rotating the hip.	Requires only one provider	Less upward force is possible. Potential injury to the provider’s forearm
Flexion adduction	One provider flexes and maximally adducts the affected hip, while the second provider applies manual pressure on the femoral head.	Allows for a controlled, steady reduction attempt	Limited data on efficacy
Foot fulcrum	Provider places patient’s foot against his or her inner ankle and places provider’s outer foot against the patient’s femoral head. Provider grasps patient’s flexed knee and leans backward.	Requires only one provider and allows for a controlled, steady reduction attempt	Potential injury to provider’s back and patient’s sciatic nerve if incorrectly performed. Risk of fall injury.
Howard	Provider grasps affected leg with both knee and hip flexed to 90 degrees, applying in-line traction, while a second provider applies lateral traction.	Allows for a slow, controlled reduction attempt	Multiple providers are needed. Limited data on efficacy.
Lateral traction	Provider grasps affected leg in extension and applies in-line traction, while a second provider applies lateral traction.	Valuable technique when the patient is unable to flex the affected hip	Multiple providers are needed. Limited data on efficacy.
Lefkowitz	Provider places his or her knee underneath the affected leg with both knee and hip flexed to 90 degrees. Provider applies a downward force on the patient’s lower leg, using the knee as a fulcrum.	Requires only one provider and allows for a controlled, steady reduction attempt	Potential to injure patient’s knee ligaments. Difficult to provide significant force for the reduction.
Captain Morgan	Provider places his or her knee underneath the affected leg with both knee and hip flexed to 90 degrees. Provider plantarflexes ankle to facilitate the reduction.	Requires only one provider and allows for a controlled, steady reduction attempt	May be more difficult in patients with longer legs.
Postgraduate Institute (PGI)	Provider gradually flexes knee to 120 degrees of flexion, then abducts to 45 degrees, and finally externally rotates until the hip reduces.	Allows for a controlled, steady reduction attempt and does not require significant force	Limited data, but appears promising.
Piggyback/rocket launcher	Provider places patient’s flexed knee over his or her shoulder and rises to a standing position	Requires only one provider and allows for a controlled, steady reduction attempt	Excess pressure on the lower leg can injure the knee ligaments.
Skoff	Patient is placed in left lateral decubitus with the leg in 100 degrees of hip flexion, 45 degrees of internal rotation, 45 degrees of adduction, and the knee bent to 90 degrees. In-line traction is applied to the leg, while another provider applies pressure to the greater tuberosity.	Allows for a controlled, steady reduction attempt	Multiple providers are needed. May be difficulty to palpate the greater tuberosity. Limited data on efficacy.
Stimson	Patient is placed prone with the affected leg 90 degrees past the end of the gurney. Downward traction is applied by the provider using either the provider’s arm or the provider’s bent knee.	Well-established. Uses gravity to facilitate the reduction	Multiple providers are needed. Difficulty to monitor the patient in the prone position. Potential for the patient to fall off the gurney.
Traction–countertraction	Patient is placed in left lateral decubitus with the leg in 100 degrees of hip flexion, 45 degrees of internal rotation, and 45 degrees of adduction. One provider applies posterior traction at the upper thigh, while a second provider applies anterior traction at the lower leg.	Allows for a controlled, steady reduction attempt. The use of bed sheets for traction allows the provider freedom to use his or her hands to facilitate the reduction.	Multiple providers are needed. Limited data on efficacy.
